# Compressive Strength, Chloride Permeability, and Freeze-Thaw Resistance of MWNT Concretes under Different Chemical Treatments

**DOI:** 10.1155/2014/572102

**Published:** 2014-07-21

**Authors:** Xingang Wang, Inkyu Rhee, Yao Wang, Yunping Xi

**Affiliations:** ^1^Department of Civil, Environmental and Architectural Engineering, University of Colorado, Boulder, CO 80309, USA; ^2^Department of Civil Engineering, Chonnam National University, Gwangju 500-757, Republic of Korea

## Abstract

This study investigated compressive strength, chloride penetration, and freeze-thaw resistance of multiwalled carbon nanotube (MWNT) concrete. More than 100 cylindrical specimens were used to assess test variables during sensitivity observations, including water-cement ratios (0.75, 0.5, and 0.4) and exposure to chemical agents (including gum arabic, propanol, ethanol, sodium polyacrylate, methylcellulose, sodium dodecyl sulfate, and silane). To determine the adequate sonication time for MWNT dispersal in water, the compressive strengths of MWNT concrete cylinders were measured after sonication times ranging from 2 to 24 minutes. The results demonstrated that the addition of MWNT can increase the compressive strength of concrete by up to 108%. However, without chemical treatment, MWNT concretes tend to have poor freeze-thaw resistance. Among the different chemical treatments, MWNT concrete treated with sodium polyacrylate has the best compressive strength, chloride resistance, and freeze-thaw durability.

## 1. Introduction

Materials containing carbon nanotubes (CNTs) tend to have superior properties, so adding CNTs to cementitious materials is expected to significantly improve material strength and stiffness. However, it can be difficult to add CNTs to cementitious materials because of the hydrophobicity of CNTs, which reduces interfacial interactions between the cement matrix and CNTs. CNTs also are prone to agglomerating or clumping due to strong van der Waals forces [1]. Therefore, more effective use of CNTs in cementitious materials will require addressing two challenges: uniform dispersion of the nanoparticles and sufficient matrix bonding [2]. Some researchers have tried to solve these issues. For example, Cwirzen et al. [2] found that treating MWNTs with polyacrylic acid can improve the compressive strength of concrete by up to 50%. Bandyopadhyaya et al. [3] found that gum arabic can improve the dispersion of CNTs in water (up to 15% weight CNT content with respect to the weight of water). Chung [4] found that the use of conventional admixtures or polymers, such as silica fume, acrylic particle dispersions, methylcellulose solution, and silane, could improve the degree of dispersion. Yu and Kwon [5] used sodium dodecyl sulfate as a surfactant to achieve good CNT dispersion: dispersion was improved when CNTs were treated with a solution of H_2_SO_4_ and HNO_3_ and uniformly dispersed into a cement paste via sonication. After CNTs were treated with polyacrylic acid and polymers, analysis via scanning electron microscope (SEM) revealed improved dispersion within a cement matrix, with the persistence of a small percentage of CNTs clusters [2]. Better results were observed when CNTs were treated with a solution of H_2_SO_4_ and HNO_3_ and uniformly dispersed into a cement paste via sonication. Both untreated and treated CNTs were dispersed homogenously, but SEM analysis revealed that treated CNTs were covered with a calcium silicate hydrate (CSH). This analysis also revealed bridging across cracks and voids, thus ensuring load transfer [6]. Similar results were obtained when the experiment was conducted in 2004 [7] and 2009 [8]. These studies also revealed that a combination of physical and chemical dispersion methods is superior to each method individually [9]. Particularly, ultrasonic stirring and chemical surfactants appear to be the most effective way of dispersing CNTs in water and cement paste. Collins et al. [10] reported that although certain chemicals can improve CNT dispersion in water, this may not necessarily increase the strength of CNT-reinforced mortar, as chemical treatments may alter the concrete's consistency. An excess of CNT also appears to act as a crack initiator in cement paste. One study assessed sonication in a mixture of CNTs in cement and isopropanol (in a ratio of 0.02 by weight) using a variety of water-cement ratios. SEM analysis revealed that these CNT composites were very porous, with CNT clustering [1, 11]. In CNTs that were mixed in aqueous solutions, grains of cement had completely hydrated without any CNTs present, disallowing any bridging and causing uneven dispersal [11, 12]. When pure CNTs were added to cement, these materials exhibited diminished strength in 7- and 14-day tests, but superior strength at 28 days, compared with ordinary Portland cement. Additionally, MWNTs demonstrated poor interfacial interaction with cement paste when pure CNTs were added to an ultra-high-performance concrete. SEM analysis of these materials revealed that CNTs were disconnected after loading (pullout). Addition of CNTs did not significantly influence compressive or tensile strength [11, 13]. When these CNT concretes were chemically treated, material properties became more consistent (with some variability still observed). When 0.06–0.42% CNTs by weight were introduced to a water suspension with added surfactant, admixtures negatively affected the compressive strength, despite the achievement of good dispersion [2, 11]. In 2008, Cwirzen et al. treated MWNTs with polyacrylic acid to aid dispersion, observing that this process also improved workability. More importantly, compressive strength was improved by 50% compared with Portland cement under loadings between 0.045 and 0.15 wt% [2]. Recently, surfactants were mixed using sonication at 0.048–0.08 wt% CNTs to improve dispersion [14]. Additionally, H_2_SO_4_ and HNO_3_ solutions were ultrasonically mixed in concrete with 0.5% wt MWNTs; this achieved a 19% compressive strength increase [7]. Most previous studies have focused on the properties of CNT-reinforced cement pastes and mortars. Because cement pastes and mortars are not commonly used in the construction industry, the present study focused on the properties of CNT-reinforced concrete. These composite materials are made of cement paste, CNTs, and fine and coarse aggregates. This investigation assessed the compressive strength and durability of CNT-reinforced concrete. The first objective was to find an optimum processing method or methods for making CNT-reinforced concrete. Different processing methods were developed based on types and dosages of dispersion agents and methods and evaluated on the basis of compressive strength. The second objective was to examine the durability of CNT-reinforced concrete: to this end, experimental tests focused on freeze-thaw resistance and chloride permeability. The findings are important to help inform possible future applications of CNT-reinforced concrete in bridge decks and overlays.

## 2. Compressive Strength of Well-Dispersed MWNT Concrete

**Figure 1:**
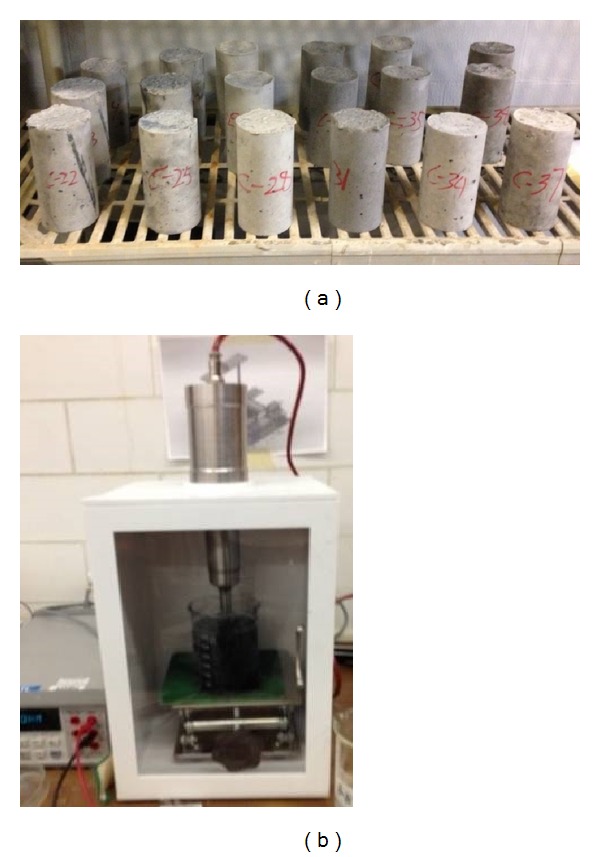
Test cylinders: (a) MWNT concrete cylinders (diameter: 2 inches, height: 4 inches); (b) sonicator apparatus.

Two different processing methods were examined to obtain a well-dispersed MWNT solution: ultrasonic dispersion and ultrasonic dispersion with different chemical treatments. MWNT fiber lengths used in this study ranged from 10 to 20 *μ*m, with an outside diameter ranging from 50 to 80 nm and an aspect ratio ranging from 125 to 400. The chemical dosage of chemicals was calculated as 0.1% of the cement weight. The chemical treatments used in experiments were (a) gum arabic, (b) propanol, (c) ethanol, (d) sodium polyacrylate, (e) methylcellulose, (f) sodium dodecyl sulfate, and (g) silane. Experiments used a sonicator with a frequency of 20 khz, a volume ranging from 300 *μ*L to 300 mL, and an output power of 450 W. To make the MWNT solutions, 300 g of water and 3 g of MWNTs were diluted with different chemicals at different weight percentages. More than 100 MWNT concrete cylinder specimens were examined to evaluate possible improvements to a control specimen, as shown in Figure 1(a). Three test parameters were used for casting MWNT test concrete cylinders (diameter: 2 inches, height: 4 inches): (a) MWNT weight percentage (ranging from 0.00 to 2.00 wt% in 0.25 wt% steps); (b) water-cement ratio (0.75, 0.5, and 0.4); and (c) chemical treatments (gum arabic, propanol, ethanol, sodium polyacrylate, methylcellulose, sodium dodecyl sulfate, and silane). All concrete cylinders were prepared using a regular concrete mix design, as shown in Table 1.

**Figure 2:**
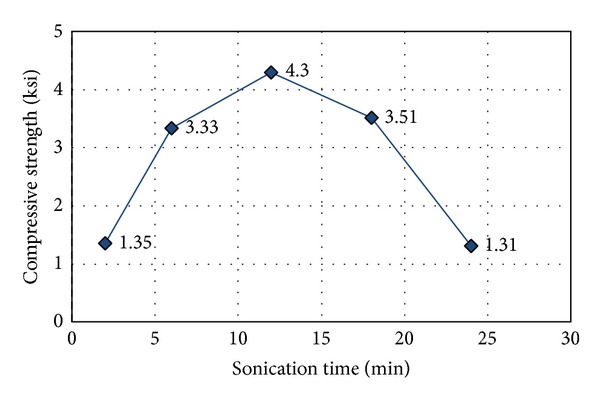
Effect of sonication time on MWNT concrete compressive strength; w/c = 0.5, 0.75 wt% of MWNT.

Prior to mixing the concrete with a well-dispersed MWNT solution, optimum sonication duration for consistent concrete mixing was determined, as shown in Figure 1(b). This step was essential to determine the workability of fresh MWNT concrete to ensure good compressive strength when hardened. Five groups of cylinders (three cylinders in each group) were cast with the same mix design, specifically w/c = 0.5 and 0.75 wt% MWNTs; these casts were exposed to different sonication durations: 2, 6, 12, 18, and 24 minutes. The compressive strength of CNT concrete increased until the sonication duration reached 12 min, after which it decreased (as shown in Figure 2). This result demonstrates that the optimum sonication time in terms of improving compressive strength is approximately 12 minutes. It appears that the hydrophobicity of MWNTs can change dramatically when MWNTs are overdispersed, so concrete workability is rapidly reduced with the increasing dispersion time. Therefore, all concrete mixing processes were sonicated for 12 minutes.

**Figure 3:**
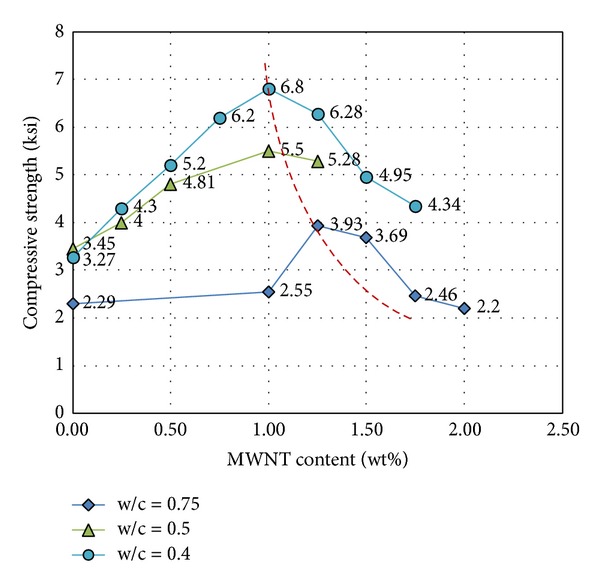
Compressive strength of MWNT concrete with different w/c and MWNT concentrations.

**Figure 4:**
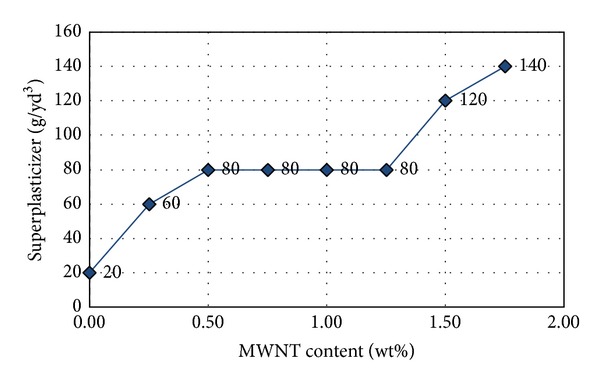
Superplasticizer dosage (g/yd^3^) at a w/c of 0.4 compared with the workability of fresh MWNT concrete mixes.

For test cylinders with a w/c of 0.75, the strength of the control specimen (MWNT 0.0 wt%) was 2.29 ksi. This finding, shown in Figure 3, suggests that the average compressive strength is not improved when the MWNT content is lower than 1.0 wt%. When the MWNT content is above 1%, the compressive strength first increases and then decreases with the increasing MWNT content. Maximum compressive strength occurred at an MWNT content of approximately 1.25%; compared with the control, the compressive strength increased by a maximum of 71.7%. For a w/c of 0.5, the workability of concrete is adequate when the MWNT content is 1.25% or less. However, when the MWNT content is greater than 1.25%, the workability of the concrete is poor. Based on this, the optimal CNT content is 1.25%. Test results indicated that the MWNT has a significant effect on the workability of fresh concrete mixtures. Figure 3 presents the average compressive strength of CNT-reinforced concrete; the average strengths at each corresponding wt% were obtained from averaging the results of three concrete cylinders. The deviation in compressive strength was approximately 10% from average for all concrete examples. Concrete compressive strength tended to increase with the increasing MWNT content: compared with the control, compressive strength increased by 59.5%. For a w/c of 0.4, different concentrations of superplasticizers were added into the concrete mixtures to improve the workability of concrete, due to its relatively low w/c ratio. With the superplasticizer addition, MWNT content ranged from 0 to 1.75%.  Figure 4 shows the relationship between superplasticizer dosage and MWNT content at a water/cement ratio of 0.4. The peak strength was 6.8 ksi at 1.0 wt%, for an increase of 108% compared with pure concrete with the same w/c ratio, as shown in Figure 3.

## 3. Sensitivity to the Addition of Different Chemical Treatments

**Figure 5:**
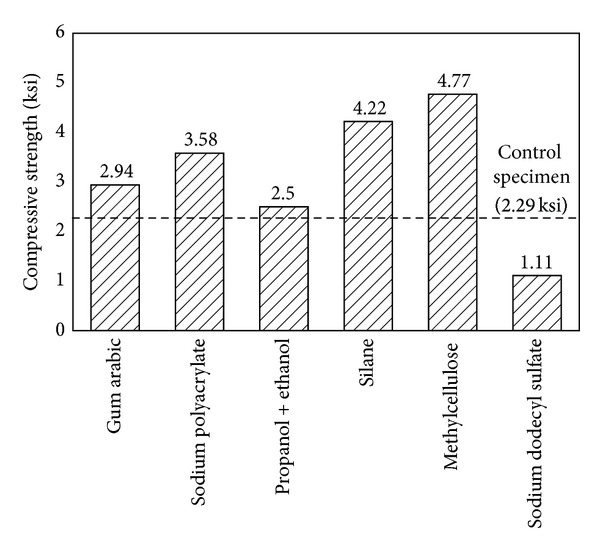
Compressive strengths of chemically treated concretes containing CNT (w/c = 0.75, MWNT = 0.75 wt%).

To determine how chemical treatments affect cement properties, aqueous solutions of CNTs were mixed using different dispersion periods, because the optimum ultrasonic period differs depending on which chemical treatment is used.  Figure 5 presents the results for various chemically treated concretes containing CNT. Some chemical treatments, such as sodium polyacrylate, methylcellulose, and silane, had positive effects. Others, such as propanol and ethanol, had poor effects. Sodium dodecyl sulfate (SDS) resulted in the lowest compressive strength over all three dispersal periods.

**Figure 6:**
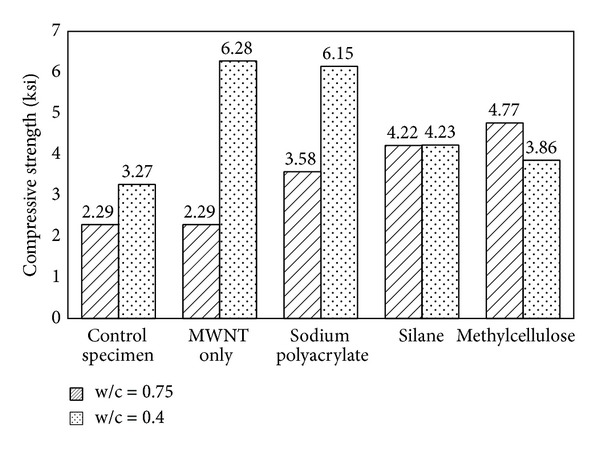
Effect of chemical treatments under two different w/c ratios.

Sodium polyacrylate, silane, and methylcellulose appear to be promising chemical treatments for improving compressive strength. SDS-treated MWNT concrete had the lowest compressive strength, which might have been the result of air entrained by SDS. In the present study, the method set out by Bandyopadhyaya et al. [3] was used to make MWNT concrete with gum arabic; however, the resultant concrete strength was only 1.74 ksi. This result indicates that this processing method, developed for MWNT cement paste and mortar, cannot be directly used for CNT concrete. The three optimal chemicals were used to make concrete with a w/c ratio of 0.4; the results revealed that the strength of chemically treated CNT concretes diminished with decreasing w/c, as illustrated in Figure 6. Compared with pure CNT concrete, all three chemically treated concretes exhibited a large increase in strength, but concretes with a w/c ratio of 0.4 did not. This finding suggests that methylcellulose and silane could help increase concrete strength at high w/c values. At low w/c ratios, the hydrophilicity of the chemical treatment might negatively affect incorporation of CNTs. Among these chemicals, sodium-polyacrylate-treated CNT concrete seems to be as strong as pure MWNT concrete. Determining the durability of these concretes will reveal whether these chemicals could be used as commercial concrete treatments.

## 4. MWNT Concrete Durability Issues

Three optimum mixes were selected based on test results for compressive strength, and CNT concrete samples were prepared for two durability tests. These three mixes all had a w/c of 0.4 and an MWNT content of 1.25 wt% but were treated with different chemicals: methylcellulose, sodium polyacrylate, or silane. These were processed by sonication and subjected to two different durability tests: a ponding test and a freeze-thaw test. MWNTs have a lower electric resistance compared with pure concrete, so MWNT concrete is more conductive. The ponding test (ASTM C1543) was used to evaluate the indirect conductivity of MWNT concrete. Concrete samples were clamped with plastic molds, sealed by epoxy, and sodium chloride solution was poured and maintained at a depth of two inches above the concrete surface at all times. After 15 days of ponding, concrete samples were drilled, and concrete powders were collected at different depths to test the chloride concentration profile. These concentration profiles were then used to determine the chloride permeability.

**Figure 7:**
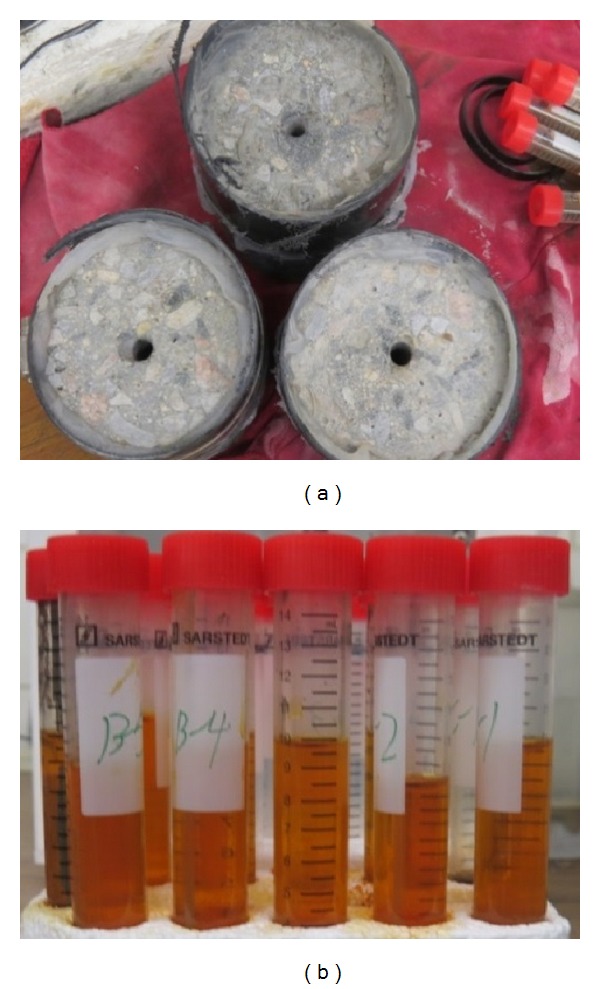
Ponding test: (a) concrete specimen with NaCl solution on top; (b) mixing concrete powder with NaCl solution.

**Figure 8:**
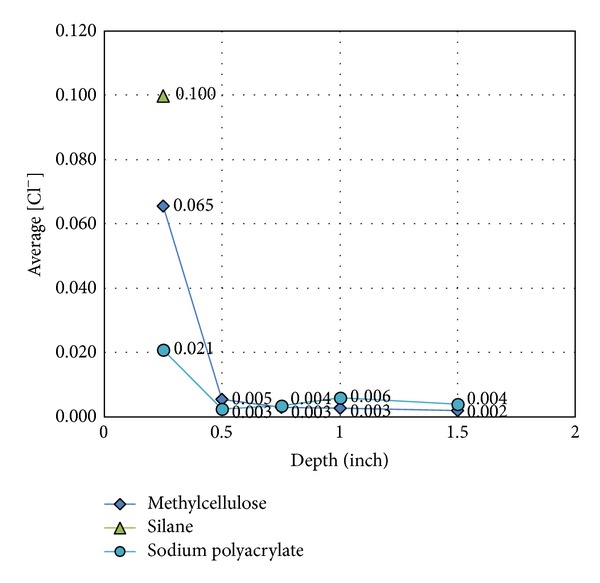
[Cl^−^] at different concrete depths.

After 15 days, the NaCl solution was discarded, and the concrete was drilled at different depths: 0.25, 0.5, 0.7, 1, 1.2, and 1.5 inches, as shown in Figure 7(a). Concrete powders were then mixed with 10 mL of chloride solution and left for 24 hours, as shown in Figure 7(b). Conductivity data were obtained after 24 hours. The results revealed that sodium-polyacrylate-treated cements had the lowest [Cl^−^] penetration at the depth of 0.25 inches. This sodium polyacrylate-CNT concrete was quite compacted, so it did not allow much [Cl^−^] penetration, making it the best chemical treatment option (see Figure 8).

The freeze-thaw test was based on ASTM C666: changes in length, weight, and pulse velocity were measured every 30 cycles. As in the other experiments, 3-inch × 6-inch specimens were surrounded by water at a depth of 1/16 inches. For each cycle, the temperature of the center of concrete was lowered from 4°C to −18°C and subsequently raised from −18°C to 4°C. The run time per cycle was approximately 2.5 hours, which is a reasonable range according to ASTM C666 (2–5 hours). An ultrasonic impulse method was used to test the relative dynamic elastic modulus. The transmit time was tested at the beginning and every 30 cycles after that. Changes in weight and length were also measured every 30 cycles. The formula for the dynamic modules is shown in (1), where *t*_*S*,0_ is the initial transmit time and *t*_*S*,*n*_ is the transmit time after *n* cycles.

(1) $$\begin{array}{c} \text{RDM}={\left(\frac{t_{S,0}}{t_{S,n}}\right)}^{2}. \end{array}$$

**Figure 9:**
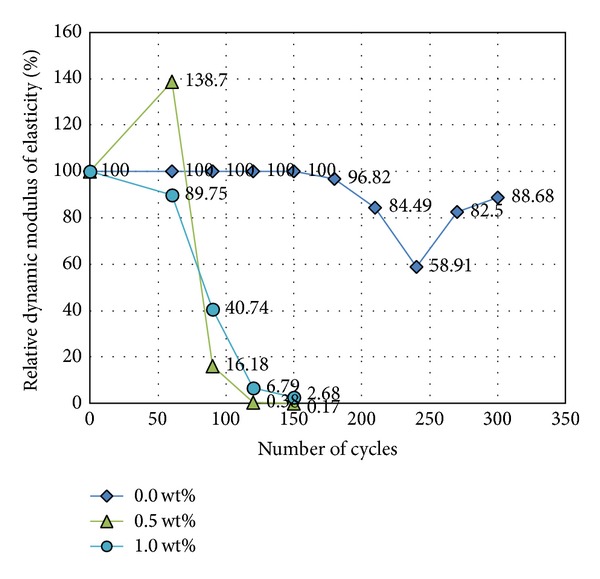
Relative dynamic elastic modulus of different concrete mixes.

Dynamic modulus results are given in Figure 9. Several specimens appeared to break after 150 cycles. Additionally, pure CNT concretes (0.5 wt% and 1.0 wt%) and CNT concretes treated with methylcellulose exhibited some scaling and a decrease in elasticity, collapsing after only 150 cycles. Pure concrete without CNTs, CNT concrete treated with silane, and CNT concrete treated with sodium polyacrylate remained undamaged after 300 cycles.

**Figure 10:**
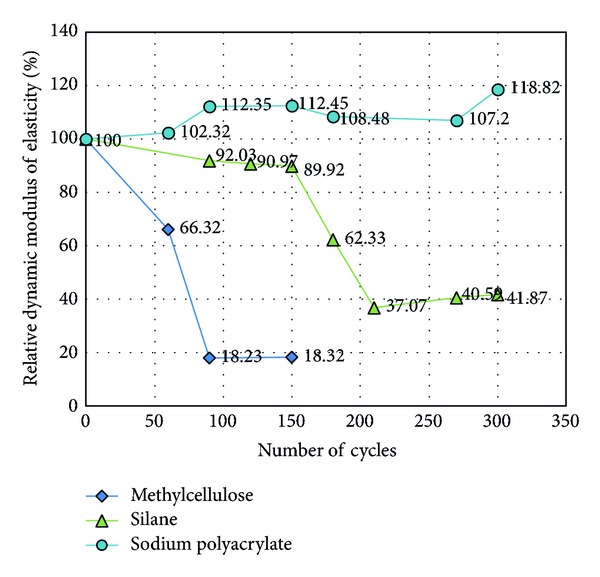
Relative dynamic elastic modulus arising from different chemical treatments.

According to ASTM C666, the concrete that fails before completing 300 cycles is classified as having poor durability. As shown in Figure 10, the surface of 0.5 wt%, 1.0 wt%, 1.5 wt%, and methylcellulose-treated concrete collapsed, and the formation of aggregates and sands could be easily observed. Silane- and sodium-polyacrylate-treated CNT concretes were the only concrete samples to remain undamaged, apart from pure concrete. In particular, the sodium-polyacrylate-treated concrete had a smooth surface and effectively resisted breaking.

**Figure 11:**
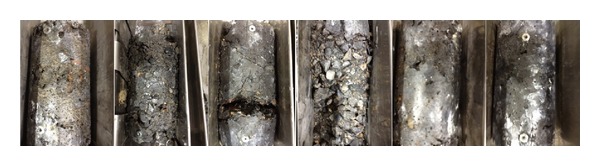
CNT concrete after 300 cycles of freezing and thawing (left to right: 0.0 wt% CNT, 0.5 wt% CNT, 1.0 wt% CNT, methylcellulose + 1.2 wt% CNT, silane + 1.2 wt% CNT, and sodium polyacrylate + 1.2 wt% CNT).

A temperature gradient occurs between the surface and the center of concrete samples, which changes the concrete strain at different depths. This means that stretching and shrinkage occur at different rates at different concrete depths, which could create cracking. Consequently, the rate of freeze-thaw damage is related to the rate of internal drainage and the rate of external water update. In this experiment, a w/c ratio of 0.4 caused concrete to have a low porosity, with a dense pore structure. The addition of CNTs also caused a dense concrete pore structure. Thus, internal drainage occurred much faster than water uptake, leading to overall shrinkage. The combined effect of these two phenomena determined the poor freeze-thaw durability for CNT concrete. Scaling on the concrete surface of the concrete decreased the dynamic modulus but increased water uptake, thereby increasing the rate of deterioration. Finally, freeze-thaw cycles generated a network of microcracks. After 300 cycles, the silane-treated CNT concrete had a much lower dynamic modulus than pure concrete. Silane-treated CNT concrete had some small defects, with some pieces falling off the surface. This result makes sense, because this concrete also had a relatively low dynamic modulus. Sodium-polyacrylate-treated concrete was very sound compared with pure concrete. Given its high compressive strength and high dynamic modulus, sodium-polyacrylate-treated concrete appears to be one of the most promising mixing options, as shown in Figure 11.

**Figure 12:**
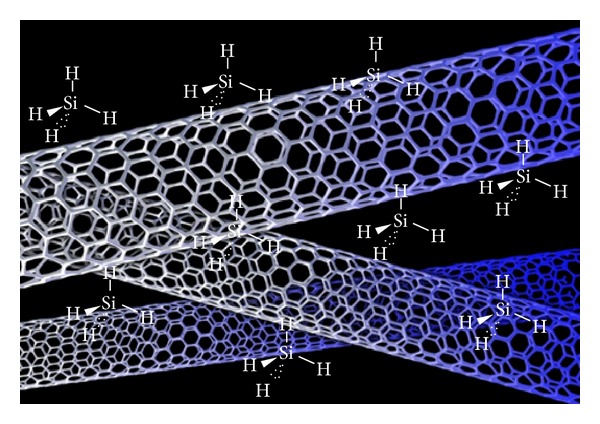
Silane (SiH_4_) helps disperse MWNT during noncovalent modification.

**Figure 13:**
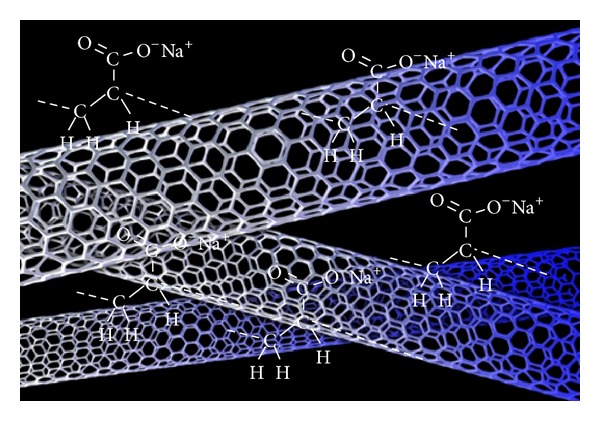
Sodium polyacrylate (C_3_H_3_NaO_2_) helps disperse MWNT during noncovalent modification.

All three chemical treatments dispersed CNTs well, producing good results for compressive strength at a water/cement ratio of 0.75, but they differed substantially in terms of durability. Silane is a silicon analogue of methane, with a chemical formula of SiH_4_. The polarity of Si–H, however, is greater than that of C–H, making its bonds more like ionic bonds. Silane also has a coupling effect, which stabilizes fibers. Methylcellulose is an ether, and Fu and Chung (1996) found that adding methylcellulose increases thermal stability. This affects the coefficient of thermal expansion (CTE) in an unstable manner, leading to substantial freeze-thaw damage [15]. They also found that methylcellulose decreases the thermal conductivity of concrete, so the temperature gradient between the surface and the center of the concrete will increase [16]. Sodium polyacrylate is a synthetic polymer composed of one repeating formula, [–CH_2_–CH(COONa)–] linked together in a long flexible chain. In terms of quantifying properties, sodium polyacrylate must absorb 100 times more water than its original weight, forming a product that resembles artificial snow. Sodium polyacrylate is a polymer with negatively charged ionic groups along its length, with accompanying positively charged sodium ions associated from the solution. Treatment methods involving silane, sodium polyacrylate, and methylcellulose differ. Methylcellulose involves covalent surface modification; in other words, it will increase the wettability of CNTs, thus reducing their tendency to agglomerate. Sodium polyacrylate and silane both involve noncovalent surface modification, which means that the hydrophobic part of the molecule is attached to MWNT sidewalls. The hydrophilic part is capable of increasing the solubility of treated CNTs.  Figure 12 shows how noncovalent modification occurs.  Figure 13 shows how sodium polyacrylate and silane help disperse CNTs in water on a microscopic scale. Both chemicals have the same hydrophobic portion: [H]^−^; the hydrophilic part of silane is [Si]^4+^, and that of sodium polyacrylate is [Na]^+^. Sodium polyacrylate is better in concrete applications because of its long chain, where hydrogen molecules will be absorbed by MWNT sidewalls. Strong van der Waals forces between hydrogen molecules and the sidewall attach this long-chain polymer more easily than the shorter, single silane molecule. Additionally, [Na]^+^ dissolves more easily in water than [Si]^4+^ does, due to the stronger polarity of [Na]^+^ compared to [Si]^4+^. These properties make sodium polyacrylate a better chemical additive to CNT concrete. Additionally, these results demonstrate that noncovalent modification is better than covalent modification for improving both the compressive strength and durability of CNT concrete.

## 5. Conclusions

Adding CNTs to concrete can significantly increase strength, by as much as 108%. With a high w/c (e.g., 0.75), the strength first increases but reaches a maximum strength at an optimal CNT concentration. This optimum CNT content depends on the w/c ratio. For w/c values of 0.4 and 0.5, the optimum CNT content is 1% by cement weight; for a w/c of 0.75, the optimum CNT content is 1.25% by cement weight. A well-dispersed CNT solution may not lead to high-strength CNT concrete. The test results demonstrated that an overdispersed CNT solution reduces the workability of CNT concrete and results in poor strength. The hydrophobic properties of CNTs can be changed dramatically, and concrete workability can be rapidly reduced with increasing dispersion time. Despite these findings, the exact causal mechanism behind these trends is not well understood. Methylcellulose, sodium polyacrylate, and silane are effective dispersion agents for CNTs; among these chemicals, treatment with sodium polyacrylate produces CNT concrete with superior freeze-thaw durability, increased compressive strength, and reduced chloride permeability.

## Figures and Tables

**Table 1:** Concrete mix designs (lb/yd^3^).

C	W	S	G	w/c	*W*_total_	a = S + G	a/*W*_total_	S/G

600	300	1,430	1,740	**0.5**	4,070	3,170	0.78	0.82

C: cement, W: water, S: sand, G: gravel, *W*_total_: the weight/yd^3^, and a: aggregates.
